# Birth-related PTSD symptoms and related factors following preterm childbirth in Turkey

**DOI:** 10.1007/s12144-022-03805-5

**Published:** 2022-10-29

**Authors:** Gözde Gökçe İsbir, Figen İnci, Burcu Kömürcü Akik, Wilson Abreu, Gill Thomson

**Affiliations:** 1 Midwifery Department, School of Health, Mersin University, Mersin, Turkey; 2 Psychiatric Nursing Department, Faculty of Zübeyde Hanım Health Sciences, Niğde Ömer Halisdemir University, Niğde, Turkey; 3 Department of Psychology, Faculty of Languages and History-Geography, Ankara University, Ankara, Turkey; 4 School of Nursing and Research Centre, CINTESIS/ESEP (Center for Research in Health Technologies and Services), University of Porto, Porto, Portugal; 5 Maternal and Infant Nutrition and Nurture Unit (MAINN), School of Health, University of Central Lancashire, Preston, UK

**Keywords:** Birth-related PTSD, Mothers, Preterm birth, Premature, Infant

## Abstract

**Objective**: To examine factors associated with birth-related post-traumatic stress disorder (PTSD) among women who had preterm birth in their last pregnancy in Turkey.**Methods**: 304 women were asked to report sociodemographic factors, perinatal factors, birth-related factors, preterm birth/premature infant characteristics, and social support factors and PTSD symptoms. Data were collected using online surveys between November 2020 and February 2021. Hierarchical multiple linear regression was used. **Results**: The prevalence of birth-related PTSD symptoms following preterm birth was 71.1*%*. Older age, the woman being positively affected by her own mother’s birth experience, not having traumatic experience in pregnancy and in the postnatal period, lower stress level after traumatic events experienced during birth, not feeling that their life/physical integrity was at risk during birth, having amniotomy, feeling psychologically well after childbirth, not being negatively affected by witnessing other parents’ happy moments with their babies in friend/family groups, the absence of infant illness and mother’s reporting higher positive interactions with healthcare team were associated with decreased likelihood of birth-related PTSD. Except for age and traumatic event in the postnatal period, all the variables explained 43*%* of the variance with a small effect size (*f*^*2*^ = 0.04). Stress level after the traumatic events experienced during labor was the strongest predictor of birth-related PTSD symptoms (*β* = 0.33). **Conclusion**: Wellbeing of mother and baby, facilitating interventions at labor, and positive communication with the healthcare team was associated with lower birth-related PTSD symptoms. The study findings highlighted on birth-related PTSD symptoms in mothers of preterm infants in Turkey.

Preterm births (births prior to 37 completed gestation weeks) are one of the most important global problems in maternal-fetal medicine and are a key cause of psychological stress for parents (Beck et al., 2010). The global prevalence of preterm labor is reported as 11.1*%*; this rate on average was 5% in developed countries, 18*%* in developing countries, and 60*%* in sub-Saharan countries (Blencowe et al., 2012). However, the inclusion of stillbirths or terminated pregnancies in these rates may cause proportional conflict (Liu et al., 2016). High-risk as well as low-risk healthy pregnancies can result in preterm birth. In studies conducted in different countries, it has been determined that preterm labor is encountered between 3.6*%* and 14.7%, with an average rate of 5*%* in low-risk healthy pregnancies (Kiserud et al., 2017).

Complications due to preterm births are very costly for the health system, and negatively affect the psychological well-being of families (Beck et al., 2010; Petrou et al., 2009). There is an association between preterm birth and maternal mortality (Geller et al., 2018; Kilpatrick et al., 2016) with between 22% and 41% of women with severe maternal morbidity having a preterm birth (Jakobsson et al., 2015). Additionally, preterm birth is one of the contributors to neonatal mortality rate in children below five years of age (Vogel et al., 2018) as well as to poorer neurodevelopmental outcomes (Johnson et al., 2015; Stålnacke et al., 2019; Yaari et al., 2018). Mothers who experience preterm birth can face challenges in maintaining their psychological well-being and adapting to their parenting role, their emotional state, self-perception and forming early attachment relationships with their infant (Trumello et al., 2018). Evidence highlights that parents of premature babies can often feel traumatised due to having a negative birth experience, or concerns of infant development or viability, leading to feelings of anxiety, fear, grief and depression, changes in appetite and sleep patterns, and social withdrawal (Baraldi et al., 2020). A review of the evidence found that 14*-79%* of women who experienced preterm birth had post-traumatic stress disorder (PTSD) symptoms (Beck & Harrison, 2017). The variation in rates is believed to be associated with the level and quality of support available. For example, research has identified that parents who have strong social support resources, have positive interpersonal relations, or have good communication with the neonatal staff have better adaptation to the traumatic experience (Koliouli & Gaudron, 2018; Lutkiewicz, 2020). Furthermore, although the importance of spousal support is known, especially in maintaining the psychological well-being of mothers, fathers of a preterm infant who experience PTSD symptoms similar to mothers, are unable to provide suitable support (Pace et al., 2020). However, preterm birth is not only a problem faced by heterosexual couples. Although the number of studies is very limited, it should be considered that preterm birth also has important consequences for individuals who do not have partners, who belongs to the LGBTQIA + community, or those who are gestational surrogates (Perkins et al., 2016; Woo et al., 2017).

Evidence has also found that PTSD symptoms can continue over time. A study by Pierrehumbert et al. (2003) found that symptoms from at least one of the four PTSD categories of symptoms (i.e. intrusion, avoidance, alterations in cognition and mood and alterations in arousal and reactivity), was observed in the first six months after delivery in 40*%* of women who had a preterm birth, and that 26*%* of these women continued to experience these symptoms until 18 months post-delivery. In another study, one-third of the PTSD symptoms seen in parents who had preterm birth experience became clinically severe in the third or fourth months after birth, then decreased over time, but continued after the 24th month in one-fifth (Pace et al., 2020). In addition, a study by Barthel et al. (2020) found that PTSD symptoms persist at a higher prevalence in the first five years in families with preterm birth experience when compared to parents with full-term birth, and recommended that families with preterm birth experience need professional support in the early postnatal period. Lastly, based on recent studies, it is claimed that the experience of trauma or the effect of that experience is “passed” somehow from one generation to the next through possibly epigenetic mechanisms (Bohacek & Mansuy, 2015; Ferguson-Smith, 2011). This highlights the impact of familial experience (Yehuda et al., 2018). Thus, we added a question to get information about the women’s perception (as positive or negative) regarding her own mother’s birth experience.

In this study, we are interested in studying factors affecting the birth-related PTSD symptoms with an integrative and comprehensive approach. Sociodemographic factors, intergenerational transmission of birth experience, pregnancy-related factors, birth-related factors, preterm birth-related factors, and social support could be evaluated as the most important variables in predicting birth-related PTSD. There are no studies that put together all the aforementioned variables and examine the joint associations with birth-related PTSD symptoms. Studies in the literature show that peripartumal (before, during and after birth) factors play a role in birth-related PTSD (Muzik et al., 2016; Vogel & Homitsky, 2020). Currently, there are a number of studies that evaluate the effects of having a preterm birth experience on women’s mental health (e.g., de Paula Eduardo et al., 2019; Cowell et al., 2021). However, little is known about what sociodemographic (e.g. age, ethnicity, marital status), clinical (birth related, e.g. having an amniotomy (the intentional rupture of the amniotic sac by an obstetrical provider), or infant related, e.g. infant health after birth), psychological (e.g. experiences of trauma, psychological wellbeing) or social factors (e.g. relationships with neonatal staff, partner) are associated with birth-related PTSD symptoms following a preterm birth. This knowledge would help provide a comprehensive understanding into what factors may predispose a woman to develop PTSD symptoms and to inform remedial actions and interventions.

This study is important in terms of being a study on preterm birth in Turkey and dealing with birth-related PTSD symptoms. This study was carried out to determine factors affecting the development of PTSD symptoms in women who had preterm birth experience in the last 10 years. Specifically, the aims of this study are; in women who had preterm birth (1) to determine the prevalence of the birth-related PTSD symptoms, (2) to understand whether women with a history of pre-term birth experience higher levels of self-reported birth-related PTSD symptoms, (3) to examine factors (e.g., sociodemographic factors, intergenerational transmission of birth experience, perinatal factors, birth-related factors, preterm birth related factors, and social support) affecting the birth-related PTSD symptoms.

## Methods

### Study design

A retrospective cross-sectional study was undertaken with women who had a preterm birth experience in the last 10 years in Turkey.

### Participants and procedure

Purposive sampling was used to target mothers who were 18 years of age and older, had given birth to a live infant who was between 22 and 37 weeks gestational age at the time of delivery (to meet the inclusion criteria for preterm birth), at least one month to ten years had elapsed since their last birth (to diminish recall bias as possible), do not have any diagnosed mental illness, their symptoms were not due to medication, substance misuse, or other illness (to prevent confounding effects on birth-related PTSD symptoms as possible), and whose native language is Turkish (to meet good reading and comprehension skills as possible). Women were asked to complete the survey using an online survey platform (forms.google.com). The link to the survey was shared through Facebook groups, WhatsApp groups, and Instagram stories by researchers (GGI and FI) and students doing an internship in the Midwifery Department at Mersin University in Turkey to reach participants who met the inclusion criteria. Data collection began on 17 November 2020 and ended on 22 February 2021. This study was approved by the Ethical Committee of the Niğde Ömer Halisdemir University (Date: 1 July 2020, No: 06). All the participants were given information about the study and online informed consent was obtained.

## Measures

### Sociodemographic and clinical information form

This form was created by the authors and comprised questions relating to sociodemographic details, PTSD symptoms, prenatal and perinatal characteristics, birth-related factors, preterm birth/premature infant characteristics, and social support characteristics of the participants (e.g., age, marital status, educational level, type of delivery, health status of the infant, interaction score with the healthcare team etc.). Additionally, a single item was used to capture intergenerational impacts of birth by asking participants to evaluate their perceptions of their mothers’ birth experience as positive or negative (*“How were you affected by your own mother’s birth experience?”*). The measures used in the study are detailed as follows:

### City birth trauma scale (City BiTS)

Birth-related PTSD symptoms were measured using the City Birth Trauma Scale (City BiTS; Ayers et al., 2018; Turkish version by Bayrı Bingöl et al., 2021); a scale consisting of 29 items evaluating PTSD symptomatology. The scale was developed to correspond with DSM-5 criteria (A [Q1-2], B-E [Q3-22], F [Q26], G [Q27-28], exclusion criteria item, whether symptoms related to illness, substance misuse, medication [Q29]) but adapted to be specific to childbirth. Criterion A (Q1-2) items are scored on a yes/no scale. DSM-5 symptoms Criteria B to E (Q3-22) are measured using 20 items that measure the frequency of symptoms for these items over the last week, rated on a four-point scale with scores from zero (not at all) to three (five or more times). The total symptom scores for Criteria B to E range from 0 to 60 and symptoms are considered present if an item is rated as one or more. Two questions (Q23-24), scored the same as Criterion B to E, identify a dissociative subtype so they are not symptoms of PTSD. The scores for Criteria F (Q26) range from zero (symptoms < 1 month/no symptoms) to two (symptoms lasting > 3 months). Criterion G items (Q27-28) are rated as yes/sometimes/no and the exclusion criteria item (Q29) is rated as yes/maybe/no. The City BiTS provides a measure of total PTSD symptoms, PTSD symptom clusters and dissociative symptoms as identified in previous research (Ayers et al., 2018; Handelzalts et al., 2018; Nakic Radoš et al., 2020). The Cronbach’s alpha in the study sample was 0.91 for the Criteria B to E [Q3-22] measured using 20 items.

## Statistical analyses

All statistical analyses were conducted using SPSS version 22, using a 0.05 significance level. Before statistical analyses, the data was checked to assess whether they fulfilled the normality assumptions. Data was normally distributed between ± 2.0 values (George & Mallery, 2010). First, participants’ sociodemographic characteristics, perinatal factors, birth-related factors, preterm birth and having a premature infant, and social support characteristics and frequency distributions were assessed. Correlations between variables were calculated using the Pearson’s coefficients (for the relationships between continuous variables with birth-related PTSD) and Spearman coefficients (for the relationships between categorical variables with birth-related PTSD) and then hierarchical multiple linear regression analyses were undertaken to assess whether and which variables are associated with birth-related PTSD. Marital status, income (all reported as middle income), and interventions during childbirth (all reported they had been exposed to interventions) were not included in the models due to their low variance in the sample. Only two single women out of 304 took part in the survey. Variables with significant correlations were entered into a forced entry hierarchical multiple regression analysis using the Stepwise method to ascertain which variables were most predictive of birth-related PTSD symptoms. Variables were entered in blocks according to the groups they belonged to (see Table1). Our theoretical approach in entering variables was to enter variables as possible as chronically. We entered demographics variables first, and then the item regarding intergenerational transmission, factors before, during, and after pregnancy, and lastly items regarding social support, respectively. Considering the birth-related PTSD symptoms as dependent variable, of the demographic variables, age was entered in the first step. The perinatal factors (planned pregnancy [yes/no], desired pregnancy [yes/no], using assisted reproductive technique use [yes/no], parity [single vs. twin and/or above], regular follow-up of pregnancy [yes/no], prenatal knowledge of premature birth [yes/no], having pregnancy-related illness during pregnancy [yes/no], participating antenatal education [yes/no], hospital stay during pregnancy and birth[yes/no], experiencing traumatic event in pregnancy [yes/no], and experiencing traumatic event in postnatal period [yes/no]) in step 2, birth-related factors (gestational week, type of birth [vaginal/cesarean], actualizing the expected/planned birth type [yes/no], interventions during labor, such as amniotomy [yes/no], enema [yes/no], continuous NST [yes/no] etc., stress level after traumatic events you experienced during birth [yes/no], feeling psychologically unwell after giving birth [yes/no], and the feeling of life/physical integrity was at risk [yes/no]) in step 3, preterm birth/premature infant characteristics (illness of infant [yes/no], perception regarding baby’s health at first sight [healthy/unhealthy], difficulties regarding preterm birth, such as seeing other babies die in NICU [yes/no], fear of not being able to take care of the baby at home after discharge [yes/no], and long separation from your baby [yes/no], being negatively affected by witnessing having tolerance seeing their other parents’ happy moments with their babies in friend/family groups [yes/no] etc., and the feeling of newborn’s life/physical was integrity at risk [yes/no]) in step 4, and social support characteristics (social support [being alone vs. having social support], interaction with healthcare team [1 to 7] and interaction with partner [1 to 7]) in step 5. Cohen’s *f*^*2*^ method was used as a measure of effect size, with *f*^*2*^ ≥ 0.02, *f*^*2*^ ≥ 0.15 and *f*^*2*^ ≥ 0.35 representing small, medium and large effect sizes, respectively (Cohen, 1988). In order to avoid problems of multicollinearity, variance inflation factors (VIF) were calculated to control for increases in the variance of the estimated regression coefficients when predictors were correlated. VIFs less than 5 were accepted (Akinwande et al., 2015).

**Table 1:** Descriptive characteristics of study sample

Sociodemographic characteristics	M ± SD or n (%)
Age (20 to 51) §	31.25 ± 5.14
18–24 vs. 25–34 vs. 35–51^**^	26 (8.6) vs. 206 (67.8) vs. 72 (23.7)
Educational level (≤ High school vs. ≥ College)	90 (29.6) vs. 214 (70.4)
Residence (City vs. Town / Village)	234 (77.0) vs. 70 (23.0)
Time elapsed since childbirth	
0 - < 1 year	149 (49.0)
1 - < 2 years	86 (28.3)
2- < 3 years	37 (12.2)
3 - < 4 years	15 (4.9)
4 - < 5 years	4 (1.3)
> 5 years	13 (4.3)
Childbirth occurred before and during the Covid-19 (Before vs. During)	185 (60.9) vs. 119 (39.1)
First time parent (Yes vs. No)	180 (59.2) vs. 124 (40.8)
**Intergenerational transmission of birth experience**	
Being influenced by own mother’s birth experience (Positive vs. Negative) §	235 (77.3) vs. 69 (22.7)
**Perinatal factors**	
Planned pregnancy (Yes vs. No)	237 (78.0) vs. 67 (22.0)
Desired pregnancy (Yes vs. No)	278 (91.4) vs. 26 (8.6)
Assisted reproductive technique (Yes vs. No)	46 (15.1) vs. 258 (84.9)
Singleton vs. Multiple birth (Yes vs. No)	57 (18.8) vs. 247 (81.3)
Regular follow-up of pregnancy (Yes vs. No)	296 (97.4) vs. 8 (2.6)
Prenatal knowledge of premature birth (Yes vs. No)	223 (73.4) vs. 81 (26.6)
Pregnancy-related illness during pregnancy (Yes vs. No)	106 (34.9) vs. 198 (65.1)
Antenatal education (Yes vs. No)	21 (6.9) vs. 283 (93.1)
Hospital stay during pregnancy and childbirth (Yes vs. No)	167 (54.9) vs. 137 (45.1)
Traumatic event in pregnancy (Yes vs. No) §	165 (54.3) vs. 139 (45.7)
Traumatic event in the postnatal period (Yes vs. No) §	163 (53.6) vs. 141 (46.4)
**Birth-related factors**	
Gestational week (22 to 36 + 6) §	30.03 ± 3.30
Type of birth (Vaginal vs. Cesarean)	56 (18.4) vs. 248 (81.6)
Expected/planned birth type (Yes vs. No)	36 (11.8) vs. 267 (88.2)
Amniotomy §	34 (11.2)
Enema	20 (6.6)
Continuous NST	191 (62.8)
Frequent vaginal examination	110 (36.2)
Urinary catheterization	127 (41.8)
Artificial pain	20 (6.6)
Stress level after traumatic events experienced during birth (0 to 5) §	4.04 (1.27)
Feeling psychologically unwell after giving birth (Yes vs. No) §	283 (93.1) vs. 21 (6.9)
Feeling that life/physical integrity is at risk (Yes vs. No) §	109 (35.9) vs. 195 (64.1)
**Preterm birth/premature infant characteristics**	
Illness of infant (Yes vs. No) §	133 (43.8) vs. 171 (56.3)
Perception regarding baby’s health at first sight (Healthy vs. Unhealthy) §	157 (51.6) vs. 147 (48.4)
Seeing other babies die in NICU (Yes vs. No) §	87 (28.6)
Witnessing the suffering of families of dead babies §	78 (25.7)
Fear of not being able to take care of the baby at home after discharge (Yes vs. No) §	182 (59.9)
Intolerance witnessing other parents’ happy moments with their babies in friend/family groups (Yes vs. No) §	113 (37.2)
Performing resuscitation with your baby right after the baby is born (Yes vs. No) §	21 (6.9)
Seeing your baby in poor health directly after birth (e.g., not breathing, bad heartbeat, purple, etc.) (Yes vs. No) §	145 (47.7)
Inability to understand the signs and sounds of technological devices that provide information about the baby (Yes vs. No) §	121 (39.8)
Long separation from your baby (Yes vs. No)	267 (87.8)
Feeling that your newborn’s life/physical integrity is at risk (Yes vs. No) §	232 (76.3) vs. 72 (23.7)
**Social support factors**	
Alone vs. having social support	11 (3.6) vs. 293 (96.4)
Interaction with healthcare team (1 to 7) §	4.97 (1.73)
Interaction with partner (1 to 7)	5.43 (1.87)

Note. M: Mean, SD: Standard deviation

§ = Variables which are significantly related to birth-related PTSD symptoms following childbirth

** The categorization of age mainly based on work by Ravaldi et al. (2018)

## Results

### Participants’ characteristics

Overall, 324 women completed the survey. Women who reported their symptoms were due to medication, substance use, or other illness, (*n* = 15), who did not specify the date of birth of their baby (*n* = 4), and who lost her baby after birth (*n* = 1) were not included in the analysis. The final sample size consisted of 304 women.

Participants ranged in age from 20 to 51 years (*M* = 31.25; *SD* = 5.14). Most women were married (*n* = 302; 99.3*%*), highly educated (≥ college; *n* = 214; 70.4*%*), and living in a city (*n* = 234, 77*%*), and all reported having middle income (my income equals to my expenses). Participants’ characteristics are reported in Table 1.

### Prevalence of birth-related PTSD symptoms following preterm childbirth

In total, 71.1*%* (*n* = 216) of women had at least one of the birth-related PTSD symptoms in the first six and more than six months after birth. One hundred and eightyfive women (60.9*%*) experienced symptoms from at least one of the birth-related PTSD symptom categories in the first six months after birth, and 31 (10.2*%*) had symptoms that continued for more than six months.

### Associations between study variables and birth-related PTSD symptoms

Correlations between PTSD and study variables are reported in Table 2.

**Table 2:** Correlations between study variables predicting birth-related PTSD

*Measure*	*1*	*2*	*3*	*4*	*5*	*6*	*7*	*8*	*9*	*10*	*11*	*12*	*13*	*14*	*15*	*16*	*17*	*18*	*19*	*20*	*21*
1. Birth-related PTSD (32.59 ± 14.82)	-	− 0.14^*^	0.17^**^	− 0.28^***^	− 0.21^***^	− 0.16^**^	0.19^**^	0.51^***^	− 0.30^***^	− 0.24^***^	− 0.24^***^	0.11^*^	− 0.18^**^	− 0.13^*^	− 0.14^*^	− 0.32^***^	− 0.12^*^	− 0.11^*^	− 0.20^**^	− 0.17^**^	− 0.13^*^
2. Age		-	− 0.00	0.10	− 0.03	− 0.02	0.04	− 0.08	0.14^*^	− 0.08	0.12^*^	− 0.03	− 0.02	0.05	− 0.10	0.09	− 0.07	− 0.04	− 0.10	− 0.10	0.07
3. Being influenced by own mother’s birth experience			-	− 0.14^*^	− 0.10	0.01	− 0.06	0.05	− 0.06	− 0.02	− 0.11	0.06	− 0.16^**^	− 0.15^**^	− 0.08	− 0.02	− 0.10	− 0.10	− 0.02	− 0.06	0.02
4. Traumatic event in pregnancy				-	0.36^***^	0.20^**^	− 0.01	− 0.22^***^	0.09	0.05	0.30^***^	− 0.16^**^	0.13	0.13^*^	0.06	0.15^*^	− 0.04	0.10	0.02	0.06	− 0.10
5. Traumatic event in the postnatal period					-	0.13^*^	− 0.11	− 0.20^***^	0.22^***^	0.16^**^	0.17^**^	− 0.16^**^	0.14^*^	0.11	0.14^*^	0.14^*^	− 0.01	0.16^**^	0.12^*^	0.15^**^	− 0.09
6. Gestational week						-	− 0.07	− 0.17^**^	0.10	0.07	0.17^**^	− 0.20^***^	0.22^***^	0.22^***^	0.08	0.04	0.00	0.20^**^	0.10	0.08	− 0.02
7. Amniotomy							-	0.12^*^	− 0.11	− 0.05	0.02	0.11^*^	− 0.11	− 0.14^*^	− 0.09	− 0.08	− 0.06	− 0.00	− 0.03	− 0.07	0.01
8. Stress level following traumatic events experienced during birth								-	− 0.29^***^	− 0.16^**^	− 0.11	0.20^***^	− 0.18^**^	− 0.12^*^	− 0.11	− 0.34^***^	− 0.12^*^	− 0.15^**^	− 0.19^**^	− 0.10	− 0.02
9. Feeling psychologically unwell after giving birth									-	0.12^*^	0.06	− 0.08	0.17^**^	0.13^*^	0.12^*^	0.16^**^	− 0.03	0.13^*^	0.17^**^	0.09	− 0.01
10. Feeling that life/physical integrity is at risk										-	0.07	− 0.02	0.15^**^	0.10	0.05	0.12^*^	0.07	0.00	− 0.02	0.17^**^	0.02
11. Illness of infant											-	− 0.26^***^	0.20^***^	0.20^**^	0.03	0.02	0.02	0.17^**^	0.11	0.09	0.10
12. Perception regarding baby’s health at first sight												-	− 0.10	− 0.16^**^	− 0.11	− 0.05	− 0.05	− 0.39^***^	− 0.21^***^	− 0.21^***^	0.04
13. Seeing other babies die in NICU													-	0.58^***^	0.18^**^	0.13^*^	0.12^*^	0.08	0.20^***^	0.08	− 0.01
14. Witnessing the suffering of families of dead babies														-	0.24^***^	0.17^**^	0.17^**^	0.15^**^	0.23^***^	0.15^**^	− 0.02
15. Fear of not being able to take care of the baby at home after discharge															-	0.26^***^	0.09	0.26^***^	0.36^***^	0.16^**^	0.04
16. Intolerance witnessing other parents’ happy moments with their babies in friend/family groups																-	0.11^*^	0.22^***^	0.20^**^	0.08	0.08
17. Performing resuscitation with your baby right after the baby is born																	-	0.08	0.07	0.03	0.00
18. Seeing your baby in poor health directly after birth (e.g., not breathing, bad heartbeat, purple, etc.)																		-	0.31^***^	0.13^*^	− 0.13^*^
19. Inability to understand the signs and sounds of technological devices that provide information about the baby																			-	0.18^**^	0.03
20. Feeling that your newborn’s life/physical integrity is at risk																				-	− 0.01
21. Interaction with healthcare team																					-

*p < 0.05, **p < 0.01, ***p < 0.001. The relationships between categorical variables with birth-related PTSD symptoms were examined using Spearman’s correlations, whereas the relationships between continuous variables and birth-related PTSD symptoms were examined using Pearson’s correlations. Of categorical variables, #3 was coded as 1 = Positive, 2 = Negative; #4, #5, #7, #9, #10, #11, #12, #13, #14, #15, #16, #17, #18, #19, and #20 were coded as 1 = Yes, 2 = No

## Predictive factors Associated with birth-related PTSD symptoms

The results of the hierarchical multiple linear regression are reported in Table 3. All VIFs were acceptable, ranging from 1.03 to 1.31 in models tested. The variables entered into the steps are shown in the Statistical analyses section above.

**Table 3:** Hierarchical multiple regression of study variables predicting birth-related PTSD

Sociodemographics	*R*	*R* ^*2*^	*Adj. R* ^*2*^	*B (SE)*	*β*	*t*	*%95 CI*	*F*
Age	0.16	0.02	0.02	-2.12 (1.24)	− 0.08	-1.71	-4.56; 0.32	7.38^**^
**Intergenerational transmission of birth experience**								
Being influenced by own mother’s birth experience	0.23	0.05	0.04	4.01 (1.59)	0.11	2.52^*^	0.88; 7.14	8.18^***^
**Perinatal characteristics**								
Traumatic event in pregnancy	0.33	0.11	0.10	-3.71 (1.51)	− 0.12	-2.46^*^	-6.67; − 0.74	12.49^***^
Traumatic event in the postnatal period	0.35	0.12	0.11	0.38 (1.47)	0.01	0.26	-2.52; 3.28	10.56^***^
**Birth-related factors**								
Stress level after traumatic events you experienced during childbirth (0 to 5)	0.56	0.32	0.30	3.84 (0.59)	0.33	6.52^***^	2.68; 4.99	27.37^***^
Feeling of life/physical integrity at risk	0.59	0.34	0.33	-4.47 (1.41)	− 0.14	-3.16^**^	-7.26; -1.69	25.90^***^
Amniotomy	0.61	0.37	0.35	6.83 (2.12)	0.15	3.22^**^	2.66; 11.00	24.58^***^
Feeling psychologically unwell after giving birth	0.62	0.38	0.36	-7.80 (2.86)	− 0.13	-2.73^**^	-13.43; -2.17	22.72^***^
**Preterm birth/premature infant characteristics**								
Intolerance seeing their happy moments with their babies in friend/family groups	0.63	0.40	0.38	-4.04 (1.46)	− 0.13	-2.77^**^	-6.90; -1.17	21.59^***^
Illness of infant	0.64	0.41	0.39	-3.52 (1.42)	− 0.12	-2.47^*^	-6.31; − 0.72	20.69^***^
**Social support factors**								
Interaction with healthcare team	0.65	0.43	0.40	− 0.95 (0.39)	− 0.11	-2.45^*^	-1.72; − 0.19	19.67^***^

Note. B = Non-standardized regression coefficients; SE = Standard error; CI = Confidence intervals; *β* = Standardized regression coefficients; R^2^ = R-square; Adj = Adjusted

*p < 0.05; **p < 0.01; ***p < 0.001

With respect to sociodemographic characteristics, older age was associated with decreased overall PTSD symptoms scores and explained 0.2*%* of variation in PTSD symptoms. As for the intergenerational transmission variable, we found that women who were affected positively by her own mother’s birth experience were associated with decreased birth-related PTSD symptoms, explaining 0.5*%* of variation in birth-related PTSD symptoms. With respect to prenatal and perinatal characteristics, not having traumatic experience in pregnancy and in the postnatal period was associated with decreased birth-related PTSD symptoms. These variables explained 11*%*, and 12*%* variation in birth-related PTSD, respectively. With respect to birth-related factors, lower stress level after traumatic events experienced during birth, not feeling that their life/physical integrity was at risk, having an amniotomy, and feeling psychologically well after birth were associated with decreased birth-related PTSD, explaining 32*%*, 34*%*, 37*%* and 38*%* variation in birth-related PTSD symptoms, respectively. In addition, with respect to preterm birth and having premature infant characteristics, not being negatively affected by witnessing other parents’ happy moments with their babies in friend/family groups and the absence of the illness in the infant were associated with decreased birth-related PTSD symptoms, explaining 40*%* and 41*%* of the variance in birth-related PTSD, respectively. Finally, with respect to social support characteristics, mothers who reported more positive interactions with the healthcare team were associated with decreased birth-related PTSD symptoms. All variables together explained 43*%* of the variance of birth-related PTSD symptoms with a small effect size (*f*^*2*^ = 0.04). It was observed that all variables contributed to the variance alone, except for age and traumatic events in the postnatal period. Considering beta values, the strongest predictor (*β* = 0.33) of birth-related PTSD symptoms was the stress level after traumatic events experienced during birth.

## Discussion

This study aimed to determine the prevalence of birth-related PTSD symptoms following preterm childbirth, the level of birth-related PTSD, and factors affecting birth-related PTSD. In this study, 71.1*%* of women with preterm birth experience had at least one PTSD symptom, 60.9*%* experienced PTSD symptoms in the first 6 months, and 10.2*%* experienced PTSD symptoms more than 6 months. This finding has links with the study by Barthel et al. (2020), demonstrating that postnatal post-traumatic stress symptoms (PTSS) and a very low birth weight (VLBW) preterm birth significantly predicted PTSS 5 years postpartum for mothers. Our findings are also similar to the studies, demonstrating the prevalence rates of elevated post-traumatic stress in mothers who gave birth prematurely ranged from 14 to 79% and our findings also confer with others in that the rates of women experiencing PTSD symptoms were higher in the first six months, followed by a rapid decrease (Beck & Harrison, 2017; Pace et al., 2020). Our findings, like others, highlight that high numbers of women experience birth-related PTSD symptoms following preterm birth. These findings highlight a need to increase awareness, knowledge, and skills of health professionals to help identify, screen and guide women for PTSD symptoms and for timely support to be provided. Supporting parents after preterm birth is critical not only for their own mental health, but also due to potential implications for their relationship with their infant (Treyvaud et al., 2019).

Regarding sociodemographic characteristics, age was found to be a risk factor, whereby younger age was associated with increased birth-related PTSD symptoms. In one study, the absence of PTSD symptoms was more likely to be observed in older women than younger ones evaluated by the Impact of Event Scale (Furuta et al., 2016). However, as this finding is thought to be related to other psychological and social related issues, such as complex needs, self-efficacy etc., and more studies are needed for the relationship between maternal age and birth-related PTSD symptoms following preterm birth.

We found that women who rated their mother’s birth experience positively had lower birth-related PTSD symptom scores. Positive ratings of mother’s birth experience and lower birth-related PTSD symptoms scores were associated. Although the underlying mechanisms behind this association are poorly understood, evidence for the intergenerational transmission of PTSD (Yehuda & Lehrner, 2018) and perinatal trauma-related consequences (Horsch & Stuijfzand, 2019) are reported. Thus, while only evaluated with a single item, our finding helps to contribute to these findings, and suggest the need for further consideration of this issue during women’s antenatal care.

Our findings identified that having a traumatic experience in pregnancy and/or postnatal period was a risk factor for birth-related PTSD symptoms following preterm birth. We also found that higher stress levels after traumatic events that experienced during birth, feeling their life/physical integrity was at risk during childbirth, not having an amniotomy, and feeling psychologically upset after birth were associated with increased birth-related PTSD symptoms following preterm birth. These findings are consistent with the studies showing that adverse birth experiences predict poor mental health outcomes, including PTSD (Ayers et al., 2018) and that women’s subjective birth experiences are the most important factor in the development of birth-related PTSD symptoms (Garthus-Niegel et al., 2013). While intervention at birth is often associated with negative outcomes, our finding that amniotomy is associated with decreased birth-related PTSD scores could be explained by women’s perception of amniotomy as an important intervention to facilitate birth and for their infant’s timely access to health care. Furthermore, regarding birth type, there was no difference in the scores on birth-related PTSD symptoms according to four different delivery modes (natural vaginal birth, instrumental/interventional vaginal birth, planned cesarean, and emergency cesarean) according to the result of the additional analysis. This finding could be evaluated as consistent with the study that showed the association between maternal morbidity and prematurity regardless of the mode of delivery (e.g., Kilpatrick et al., 2016).

Regarding preterm birth/premature infant characteristics, not being negatively affected by witnessing other parents’ happy moments with their babies in friend/family groups and the absence of infant illness were associated with decreased birth-related PTSD. Regarding the association between not being negatively affected by witnessing other parents’ happy moments with their babies in friend/family groups and birth-related PTSD. Although, it is hard to define whether these are the result or cause of the birth-related PTSD, based on our results, it could be concluded that it would be good for mothers to be in environments with parent-infant relationships where they feel their own selves positive, supported and well received. Thus, more studies are needed. In regard to the association between the absence of infant illness and decreased birth-related PTSD symptoms, this was in line with a study by Chan et al. (2020), demonstrating thate birth-related PTSD the relative risk for the delivered with newborn medical complications resulting in newborn admission to the neonatal intensive care unit (NICU) and/or prematurity was higher than the healthy group. Our results are also consistent with the studies indicating that parents are more likely to feel emotionally close to their infants when their infant is improving (Flacking et al., 2016; Thomson et al., 2020).

Women with a history of preterm birth could benefit from social support on an ongoing basis in their prenatal care as well as during birth and postpartum. For example, social support was identified as the most important factor inthe development of maternal competence in mothers of late preterm infants (Baker et al., 2013). Furthermore, in a recent retrospective, case control study, according to women who have the history of preterm birth, with chronic stress and with moderate to high support from fathers, women with chronic stress and with low partner support and had an increased risk of preterm birth (Ghosh et al., 2010). We had conceptualized social support in three parts by focusing mostly on the postpartum period: having social support in the postpartum period, interaction with the healthcare team, and interaction with the partner (without indicating a definite time interval). However, only interaction with healthcare team had a predictor role in birth-related PTSD symptoms. Specifically, with respect to social support characteristics, mothers who reported more positive interaction with the healthcare team were less likely to report PTSD symptoms. Most studies showed the primary importance of the healthcare team to parent-infant wellbeing. For example, a study provided evidence that collaborative decision-making between mother and healthcare providers in the care of the infant helped to reduce maternal stress (Beck & Harrison, 2017). It is crucial that improving the psychological well-being of the family as a whole will have a direct or indirect positive effect on mother-baby-family health. A multilayered approach is suggested and includes layers of individual, psychological and psychosocial support, peer-to-peer support, and family centered care (Treyvaud et al., 2019).

## Limitations and future directions

The results of this study need to be evaluated in light of several limitations. First, the cross-sectional design of the research means that the causality of the relationships investigated cannot be determined. However, it is one of the valid and reliable methods to examine life events that experienced before. Future studies should use a longitudinal design in order to analyze the possible predictors over time. Second, our sampling procedures (i.e., data collection via web-based survey) might have captured mothers who were more digitally proficient. In addition, self-recruitment via the web might lead to an unrepresentative sample. Third, participants’ age range (20–51 years) was very large and childbirth care and medical practices, such as the advent of family centered care, have changed in recent years in Turkey. Fourth, the socioeconomic status of the participants (all reported as middle income) was very narrow, and means that the sample is unlikely to be representative. Thus, analyzing the role of age and socioeconomic status in greater detail in larger samples would be necessary in future studies. Additionally, although the time elapsed after birth did not have a significant effect on birth-related PTSD symptoms, it would be useful to consider this in more depth by including more participants at different time intervals. Further mixed-methods studies using quantitative and qualitative evidence may help shed light on key moderating and mediating variables that predict and mitigate against PTSD onset. Fifth, having a wide range of numbers in perinatal factors (e.g., regular follow-up of pregnancy) could be evaluated as another limitation of the study due to the possibility of undermining equal variances assumptions. Furthermore, excluding criteria that not have any diagnosed mental illness may not likely account for existing mental illnesses that are not reported. This may lead to non-differential misclassification which may deem the results conservative. Sixth, women who experience punitive social consequences associated with a lack of social support or abusive relationships may be at greater risk for perinatal PTSD. Therefore, it would be beneficial to include variables such as domestic violence and emotional abuse in future studies. Finally, in this study, the total symptom scores of PTSD were used. However, evaluating which clusters of PTSD symptoms are most strongly associated with which factors would be a suggestion for future studies.

## Conclusion

Despite the limitations, this study has highlighted key factors that may protect against mothers developing PTSD symptoms following a premature infant; namely older age, the woman having positive perceptions of her own mother’s birth experience, not having traumatic experience in pregnancy and in the postnatal period, lower stress level after traumatic events experienced during birth, not feeling of life/physical integrity at risk, amniotomy, feeling psychologically well after birth, having tolerance seeing their happy moments with their babies in friend/family groups, the absence of the illness in the infant and mother’s higher interaction with healthcare team were associated with decreased birth-related PTSD symptoms.

The findings from this study have important implications for public health and clinical interventions. This study has shown once again that the routine screening of birth-related PTSD is crucial for maternal and infant health. It has great importance for women to have good conditions both during pregnancy and in the postpartum period. Giving place the constructs which contribute to diminishing stress level in interventions is one of the significant implications of the current study. Considering the feeling that their life/physical integrity was at risk during birth and the role of stress level after the traumatic events during labor on birth-related PTSD symptoms, a respectful, humanized, consented and positive maternity care should be given to women. Based on the association between higher positive interactions with healthcare team and decreased likelihood of birth-related PTSD, we may conclude that training for neonatal nurses, midwives, obstetricians, gynecologists, psychologists, psychiatrists, and the wider team working in postpartum health is an inevitable implication. Lastly, approaches that contribute to improving psychological well-being in perinatal processes may be protective for birth-related PTSD symptoms following preterm birth, but this needs to be examined further.

Evaluation of birth-related PTSD symptoms in the postpartum period is crucial for perinatal mental health. For this reason, in the postpartum period, professionals (neonatal nurses, midwives, obstetricians, gynecologists, psychologists, psychiatrists, and those who are working in postpartum health) should evaluate women’s symptoms regarding birth-related PTSD with the valid and reliable measurement tools and clinical evaluation, guide them in realizing usual and unusual emotions, cognitions and behaviors following birth, guide them in the process of dealing with problematic issues if any, and offer counseling to women about birth-related PTSD.

Finally, multidisciplinary, multicentric and cross-national studies on this topic are needed, using robust methods and representative samples.
